# Fremy’s Salt-Mediated Oxidative Addition. A New Approach in the Total Synthesis of Naturally Dipetalolactone and Its Immunomodulatory Activity

**DOI:** 10.3390/molecules180911485

**Published:** 2013-09-16

**Authors:** Yasser Selim, Nabil Ouf, Mohamed Sakran

**Affiliations:** 1 Faculty of Specific Education, Zagazig University, Zagazig 44519, Egypt; 2 Department of Chemistry and Biochemistry, Faculty of Science, Tabuk University, Tabuk PO Box 699, Saudi Arabia; 3 Department of Chemistry, Faculty of Science, Zagazig University, Zagazig 44519, Egypt; 4 Department of Chemistry, Faculty of Science, Tanta University, Tanta 31527, Egypt

**Keywords:** coumarins, dipyranocoumarin, dipetalolactone, Fremy’s salt

## Abstract

The structure of the natural dipyranocoumarin dipetalolactone has been confirmed by an unambiguous synthetic route from resorcinol. This sequence was initiated by a pyran ring formation step which introduced the 3-chloro-3-methylbut-1-yne moiety. Then, the expected product undergoes a Fremy’s salt-meditated oxidative addition followed by ring closure to yield dipetalolactone. Dipetalolactone was also found to have immunological activity in a mouse carcinoma S180-bearing mice cell line.

## 1. Introduction

Dipetalolactone is a natural product dipyranocoumarin isolated from different plants like *Zanthoxylum dipetalum*, *Metrodorea flavido* and *Diplolaene mollis* (Rutaceae) [1]. The dipyranocoumarins, a group of natural products from several tropical plants of the genus Calophyllum are characterized by coumarin, chromene and chromane ring systems [2]. Biologically, dipyranocoumarins are very useful and many of them have exhibited anti HIV, antibacterial, antitumor, vasodilator (in coronary vessels) and anticoagulant activities [3]. It was long noted that most coumarins are free from toxic side effects and may be given for years without side effects; overdoses, however, may causes hemorrhages [3]. Coumarins are widespread in the Angiosperms but they are rather rare in Gymnosperms and lower plants. They present great structural variety, especially in the Apiaceae and Rutaceae, and are additionally found in many other plants families like the Asteraceae, Poaceae and Rubiaceae [4]. The family Rutaceae belongs to the order Rutales characterized by the occurrence of coumarins in all its families. Coumarins, although very frequent in the family as a whole, are confined to four sub-families (Aurantioideae, Flindersioideae, Toddalioideae and Rutoideae). The genus *Zanthoxylum* in the subfamily Rutoideae is characterized by the presence of different types of coumarins (simple, linear, dihydrofurocoumarins, furocoumarins and pyranocoumarins). The linear and angular dihydrofurocoumarins and precursors have been identified in several species of the genus, but angular dihydrofurocoumarins are not common in other species of the family Rutaceae, so it can be of chemotaxonomic value for the genus *Zanthoxylum* [5].

## 2. Results and Discussion

### 2.1. Chemistry

**Scheme 1:**
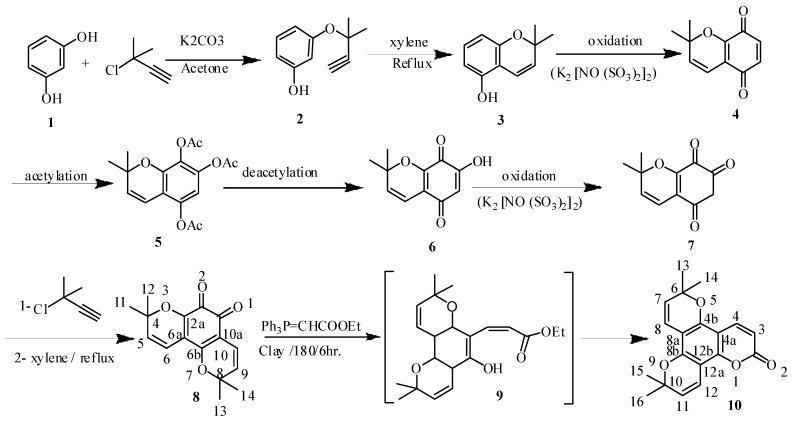
Total synthesis of natural dipetalolactone.

Previously dipetalolactone has been prepared from 5,7-dihydroxycoumarin [6]. In the context of this work, we have synthesized dipetalolactone starting from resorcinol using Fremy’s salt as meditating oxidizing agent. To our knowledge, except for the reaction of compound **1** with 3-chloro-3-methylbut-1-yne [6], there has not been any work in the same direction. Our studies were initiated by the reaction of resorcinol **1** and 3-chloro-3-methyl but-1-yne, which introduces the 3-methylbut-1-enyl group exclusively and in high yield at the OH of C1 to form 3-(2-methylbut-3-yn-2-yloxy)phenol (**2**) followed by reflux with xylene for 8 h to give pyran ring **3** (Scheme 1). The ^1^H-NMR of compound **3** indicates the presence of five aromatic protons at δ 6.1 (d, 1H, *J =* 9.1 Hz, H-3), 6.8 (d, 1H, *J =* 9.4 Hz, H-4), 6.22 (d, 1H, *J =* 8.1 Hz, H-6), 7.1 (m, 1H, *J =* 8.4 Hz, H-7) and 6.13 (d, 1H, *J =* 8.5 Hz, H-8). Further the ^1^H-NMR spectrum displayed signals of two methyl at δ 1.55 (s, 3H) and 1.65 (s, 3H). Compound **3** was oxidized when a solution of **3** is treated with a solution of KH_2_PO_4_ in water and (KSO_3_)_2_NO at 0 °C to give 2,2-dimethyl 2-H-chromene-5,8-dione (**4**). Fremy’s salt oxidizes most phenols to *p*-quinones when there is no *Para*-substituent, whereby oxygen incorporation occurs exclusively from the oxygen of Fremy’s salt on the phenol group. Compound **4** was characterized by its ^13^C-NMR spectrum which revealed the presence of two signals at δ 185.5 (C-1) and 181.7 (C-2) respectively, which indicated the presence of a *Para*-diketone structure. The quinoid structure constitutes one of the most interesting classes of compounds in Organic Chemistry [7]. The chemistry of quinones is largely dependent on the substituent being either on the quinonic or on adjacent rings. This is reflected in their chemical reactivity, especially in heterocyclic quinones [8]. Hydroxylated quinones that have one or more hydroxy groups attached directly to the quinone moiety are found in Nature in great variety. A method of broad applicability for the preparation of the hydroxy quinone moiety is through Thiele-Winter acetoxylation. The method involves the reaction of 1,4- or 1,2-quinone derivatives with acetic anhydride. Thus, the conversion of the *Para*-quinoid structure to the corresponding triacetate derivative occurred by treating it with acetic anhydride in dioxane [9,10] to give the expected product **5**. The triacetoxy derivatives—isolated in fair to excellent yields—are hydrolyzed to the corresponding hydroxyhydroquinone derivatives under either acidic or basic conditions. The latter, as a rule without isolation, are then oxidized to the desired hydroxy quinone compounds. In many cases, especially under basic conditions, the oxidation also proceeds with atmospheric oxygen [9]. Treatment of compound **5** with hydrochloric acid in the presence of ferric chloride gave the expected *ortho*-hydroxy quinone structure **6** in 88% yield. The di pyrano derivative **7** was also obtained by refluxing **6** with 3-chloro-3-methylbut-1-yne, followed by ring closure by refluxing the product in dry xylene to give compound **8**. Compound **7** was characterized by ^1^H- and ^13^C-NMR spectroscopy which revealed the presence of signals at δ 4.07 as a sharp singlet, *i.e.*, that is flanked between two ketone groups and two aromatic protons at δ 5.84, and the absence of signals of the hydroxyl group proton at 6.82. Also, the IR spectrum showed the absence of a hydroxyl group peak, thus confirming the formation of 7-hydroxyl-2,2-dimethyl 2-H-chromene-5,7,8(6H)-trione (**7**). Finally the dipyranocoumarin was prepared by Wittig reaction in the presence of clay (montmorillonite KSF) and compared with an authentic sample of dipetalolactone [6]. The UV absorption in ethanol exhibited peaks at λ_max_^Me^^oH^: 282 (log ε 3.75), 232 (log ε 3.97) and 211 (log ε 4.27) characteristic of dipyranocoumarins. The IR spectral band at 1,720 cm^−1^ indicates the presence of a δ-lactone. It was also characterized by its ^1^H- and ^13^C-NMR spectra which revealed the absence of acetyl signals, thus confirming the cyclization and formation of dipetalolactone (**10**). 

### 2.2. Biological Activity

#### 2.2.1. Effects of Compound **10** on Body and Tumor Weights of S180-Bearing Mice

All the tumor-injected mice survived following the treatments with water or any of the four doses of compound **10** until they were sacrificed for analysis of immunological features. When the tumor masses were removed, we observed that the animals treated with compound **10** had no significant changes in body and tumor weight (Table 1, *p* > 0.05), but showed lower tumor growth to a certain extent compared with the S180 control group. Higher concentrations appeared to show more effective inhibition. Complete regression of tumor was not observed in any group.

**Table 1:** Effects of compound **10** on body and tumor weights of S180-bearing mice.

Group	Concentration (mg/kg)	Body weight (g) at begin	Body weight (g) at end	Tumor weight (g)	Inhibition (%)
Normal control		25.5 ± 1.5	33.4 ± 3.55		-
S180 control		25.8 ± 1.35	32.6 ± 3.42	4.6 ± 1.2	-
Compound **10**	50	25.6 ± 1.44	32.8 ± 2.55	4.22 ± 1.11	2.2
	100	25.1 ± 1.12	33.0 ± 3.22	3.32 ± 0.85	6.1
	250	25.7 ± 1.4	33.8 ± 3.24	3.77 ± 0.92	12.5
	500	25.33 ± 1.35	33.4 ± 3.15	3.66 ± 0.62	22

#### 2.2.2. Effects of Compound **10** on Immune Functions of S180-Bearing Mice

##### 2.2.2.1. Effects of Compound **10** Extract on Humoral Immune Function

The effect of compound **10** on humoral immune function was estimated by measuring quantitative hemolysis of sheep red blood cells *in vivo* (Table 2). All dose treatments could markedly increase the antibodies secreted by spleen cells in mice, where 500 mg/kg dose was the best as a typical immunostimulator was used.

**Table 2:** Determination of LD_50_ of compound **10** given i.p. in adult mice.

Group	Concentration (mg/kg)	Quantitative hemolysis of sheep red blood cells (HC_50_)	Lymphocyte proliferation (cpm)	NK cytotoxic activity (%)	Phagocytosis rate （%）
Normal control	–	450 ± 24.1 ***	12,530 ± 4,121 **	58.4 ± 1.22 *	43.1 ± 2.15 ***
S180 control	–	60.11 ± 3.32	2,822 ± 881	48.6 ± 1.33	35.0 ± 3.31
Compound **10**	50	120 ± 9.8 ***	3,251 ± 1,161	50.2 ± 2.51	37.8 ± 2.31 *
	100	221 ± 10.4 ***	5,322 ± 1,131 **	55.8 ± 2.64 ***	39.9 ± 3.55 ***
	250	232 ± 11.2 ***	6,455 ± 1,228 *	56.6 ± 2.55 ***	41.12 ± 2.34 **
	500	243 ± 10.5 ***	7,220 ± 2,122 *	58.6 ± 3.1 ***	42.3 ± 3.21 **

Values are mean ± S.D. of 10 mice; * Significantly different from S180 control group at *p* < 0.05; ** Significantly different from S180; *** Significantly different from S180 control group at *p* < 0.001.

##### 2.2.2.2. Effects of Compound **10** on Cellular Immune Function

The effect of compound **10** on cellular immune function was estimated by measuring lymphocyte proliferation and Natural Killer (NK) cell cytotoxicity *in vivo*. In the assay, spleen lymphocyte proliferation and NK activity were significantly decreased in the S180 control group. Compound **10** at doses at 100, 250 and 500 mg/kg demonstrated remarkable spleen lymphocyte proliferation stimulation and increased NK activity, whereas the dose of 50 mg/kg did not (Table 2). In the NK cell cytotoxicity assay, additionally, the same doses of compound **10** could significantly regulate cellular immunity close to a normal level. 

##### 2.2.2.3. Effects of Compound **10** on Nonspecific Immune Function

This effect of compound **10** was estimated by measuring the phagocytic activity of peritoneal macrophages *in vivo*. Administration of compound **10** could significantly enhance the phagocytic activity of peritoneal macrophages at all doses compared to the untreated group (Table 2). The doses at 100, 250 mg/kg and 500 mg/kg showed the most effective activity, which was close to that of the normal control.

## 3. Experimental

### 3.1. General

All melting points were taken on an Electro thermal IA9000 series digital melting point apparatus. Elemental analysis data were obtained from the Micro analytical unit, Cairo University, Cairo. Egypt. The IR spectra (KBr) were recorded on the Elmer model 1430 spectrophotometer. ^1^H- and ^13^C-NMR spectra were recorded in CDCl_3_ on a Bruker Avance DRX-500 spectrometer (^1^H at 500 MHz and ^13^C at 125 MHz). ESIMS and HRESIMS experiments were performed using a Micromass Q-TOF (Manchester, UK). TLC was carried out on precoated silica gel 60 F_254_ (Merck, Munich, Germany) and spots were visualized by UV lamb. Column chromatography was carried out on silica gel 60 (63–200 μm, Merck).

### 3.2. 3-(2-Methylbutyl-3-yn-2-yloxy)phenol (**2**)

To a solution of resorcinol (0.01 mol) and potassium carbonate (0.01 mol) in acetone (30 mL), 3-chloro-2-methylbut-1-yne (0.01 mol) was added drop wise under nitrogen. The reaction mixture was heated and stirred at 70 °C for 72 h and monitored by TLC. After cooling to room temperature the solution was poured in water (100 mL) then acidified with dilute HCl (5%) to remove the residue of K_2_CO_3_ and extracted by diethyl ether (4 × 15 mL). The mixture was dried over anhydrous sodium sulphate, filtered and then the solvent was evaporated and the crude product was purified by column chromatography over silica gel (100–200 mesh) and eluted with mixtures of hexane/ethyl acetate (1:1 v/v) as eluent to give compound **2**, which was crystallized from dichloromethane/hexane as solvent system to give pale white crystals (40%) with mp 160–162 °C; IR (KBr) *ν*_max_ cm^−1^: 3,540, 3,352, 1,335, 1,119, 921, 849; ^1^H-NMR (CDCl_3_): δ 7.18 (Ar-1H, m, *J =* 9.2 Hz, H-5), 6.58 (Ar-1H, m, *J =* 9.1 Hz, H-4), 6.51 (Ar-1H, m, *J =* 8.9 Hz, H-2,6), 3.51 (1H, s, ≡CH), 1.69 (6H, s, 2CH_3_); ^13^C-NMR (CDCl_3_): 160.1 (C-3), 157.4 (C-1), 130.5 (C-5), 107.8 (C-4), 102.7 (C-2), 85.5 (C≡CH), 81.7 [C(CH_3_)_2_], 75.4 (≡CH), δ 29.2 (2CH_3_); EIMS (C_11_H_12_O_2_) (% int.) *m/z* 176 (100), 205 [M–H]^+^ (25), 190 [M–OH]^+^ (21), 176 [M–2CH_3_]^+^ (8) and 107 [M–HC≡C−C(CH_3_)_2_−O]^+^ (12); Analysis: calcd. %C, 74.98; %H, 6.83; %O, 18.18. Found: %C 74.88; %H, 6.81; %O 18.09.

### 3.3. 2,2-Dimethyl-2H-chromen-5-ol (**3**)

Compound **2** (0.01 mole) was boiled in dry xylene for 8 h under a nitrogen atmosphere, the reaction mixture was cooled, and then extracted with dichloromethane. The residue after removing the solvent was subjected to column chromatography over silica gel (800 g) and eluted with mixtures of hexane/ethyl acetate (1:2 v/v) as eluent to produce compound **3**, which was crystallized from ethanol to give white crystals (60%) with mp 155–156 °C; IR (KBr) *ν*_max_ cm^−1^: 3,540, 3,352, 1,335, 1,129, 931, 841; ^1^H-NMR (CDCl_3_): δ 11.2 (s, OH exchangeable with D_2_O), 7.13 (Ar-1H, m, H-7), 6.86 (Ar-1H, m, *J =* 9.2 Hz, H-4), 6.55 (Ar-1H, d, *J =* 8.9 Hz, H-8), 6.31 (Ar-1H, d, *J =* 8.9 Hz, H-6), 5.91 (Ar-1H, d, *J =* 9.1 Hz, H-3), 1.59 (3H, s, 2CH_3_); ^13^C-NMR (CDCl_3_): δ 156.7 (C-5), 155.5 (C-8a), 130.4 (C-7), 128.5 (C-3), 116.2 (C-4), 109.8 (C-4a), 109.7 (C-6), 106.5 (C-8), 85.7 (C-2), 28.5 (2CH_3_, C-9,10); EIMS (C_11_H_12_O_2_) (% int.) *m/z* 176 (100), 175 [M–H]^+^ (32), 160 [M–OH]^+^ (21) and 146 [M–2CH_3_]^+^ (12); Analysis: calcd. %C, 74.96; % H, 6.85; %O, 18.19. Found: %C 74.91; %H, 6.82; %O 18.12.

### 3.4. 2,2-Dimethyl-2H-chromene-5,8-dione (**4**)

To a solution of KH_2_PO_4_ (10 g) in water (200 mL) was mechanically stirred in a 2 L round bottom flask and ice (200 g) was added compound **3** (0.01 mol). The flask was cooled in an ice-ethanol mixture and (KSO_3_)_2_NO (6 g, 22.4 mmol) added, followed by a solution of 4-(methylmercapto)phenol (1.0 g, 7.1 mmol) in diethyl ether (20 cm^3^). After 1 min the solution turned orange and after 5 min dark red. After 1 h the mixture was evaporated at 20 °C to remove the diethyl ether and extracted with chloroform (3 × 50 cm^3^). Evaporation gave a crimson solid (0.95 g, 86% yields) with mp 180–181 °C; IR (KBr) *ν*_max_ cm^−1^: 1,750, 1,735, 1,343, 1,139, 951, 841; ^1^H-NMR (CDCl_3_): δ 6.93 (Ar-1H, d, H-6,7), 6.44 (Ar-1H, s, H-4), 5.81 (Ar-1H, d, *J =* 9.2 Hz, H-3), 1.42 (3H, s, 2CH_3_); ^13^C-NMR (CDCl_3_): δ 185.5 (C-5), 181.7 (C-8), 150.4 (C-8a), 136.2 (C-6), 135.5 (C-7), 132.8 (C-3), 119.7 (C-4a), 116.5 (C-4), 85.9 (C-2), 28.7 (2CH_3_, C-9,10); EIMS (C_11_H_10_O_3_) (% int.) *m/z* 190 (100), 175 [M–1CH_3_] ^+^ (22), 160 [M–2CH_3_] ^+^ (9), 162 [M–CO] (41); Analysis: calcd. %C, 69.46; %H, 5.30; %O, 25.22. Found: %C 69.41; %H, 5.22; %O 25.12.

### 3.5. 2,2-Dimethyl-2H-chromene-5,7,8-triyltriacetate (**5**)

Acetylation was carried out following literature method [5]. In brief, compound **4** (0.01 mol) was dissolved in refluxing *p*-dioxane (200 mL) and acetic anhydride (0.03 mol) for 8 h. After cooling, the acetylated compound solution was concentrated and dried under reduced pressure. The crude acetylated lignin was dissolved in chloroform (3 mL) and the solution was added with stirring to diethyl ether. The precipitated acetate was centrifuged, collected, and dried under high vacuum for 24 h. The precipitate formed after cooling the mixture on ice water solution was recrystallized from ethanol give compound **5** as white crystals (0.61 g, 50%) with mp 277–279 °C. IR (KBr) *ν*_max_ cm^−1^: 1,763, 1,750 and 1,710 (3 C=O ester), 1,576, 1,345, 1,129, 911, 849; ^1^H-NMR (CDCl_3_): δ 6.88 (Ar-1H, d, *J =* 9.9 Hz, H-4), 6.42 (Ar-1H, d, *J =* 9.1 Hz, H-6), 5.88 (Ar-1H, d, *J =* 8.5 Hz, H-3), 2.28, 2.2, 2.1 (s, 9H, 3 CH_3_CH_2_O), 1.49 (6H, s, 2CH_3_); ^13^C-NMR (CDCl_3_): δ 169.1 (3C=O), 153.2 (C-8a), 147.2 (C-5), 144.2 (C-7), 130.1 (C-8), 127.4 (C-3), 114.1 (C-4a), 116.5 (C-4), 103.5 (C-6), 86.7 (C-2), 28.2 (2CH_3_, C-9,10), 20.5 (3CH_3_-ester); EIMS (C_17_H_18_O_7_) (% int.) *m/z* 334 (100), 319 [M–1CH_3_] ^+^ (34), 304 [M–2CH_3_] ^+^ (21) and 199 [M–3OAc] (14)”; Analysis: calcd. %C, 61.08; %H, 5.43; %O, 31.48. Found: %C 60.88; %H, 5.31; %O 31.59.

### 3.6. 7-Hydroxyl-2,2-dimethyl-2H-chromene-5,8-dione (**6**)

Compound **6** was obtained by dissolving (0.1 mol) of compound **5** in 10% NaOH solution, then by addition of HCl, we obtain a precipitate, which is filtered off , then transferred into a conical flask and water (30 mL) is added. The mixture is brought to its boiling point, FeCl_3_ (1.5 g) is added and boiled for 10 min, the hot solution is filtered, washed with boiling water and he precipitate formed dried in air. Recrystallization from toluene gives brown crystals (40%) with mp 254–255 °C; UV (MeOH) *λ*_max_: 331 (log ε 3.67), 292 (log ε 4.07), 262 (log ε 4.45); IR (KBr) *ν*_max_ cm^−1^: 3,340, 1,752, 1,722, 1,576, 1,345, 1,129, 911, 849; ^1^H-NMR (CDCl_3_): δ 16.2 (1H, s, 1OH), 6.83 (Ar-1H, d, *J =* 9.1 Hz, H-2), 6.42 (Ar-1H, d, *J =* 9.2 Hz, H-8), 6.81 (Ar-1H, s, H-6), 6.41 (Ar-1H, d, *J* = 9.2 Hz, H-4), 5.81 (Ar-1H, d, *J =* 8.4 Hz, H-3), 1.39 (6H, s, H-10,11); ^13^C-NMR (CDCl_3_): δ 182.5 (C-5), 196.2 (C-8), 176.5 (C-7), 141.2 (C-8a), 126.5 (C-3), 117.4 (C-4a), 115.5 (C-4), 109.5 (C-6), 86.7 (C-2), 29.5 (2CH_3_, C-9,10); EIMS (C_11_H_10_O_4_) (% int.) *m/z* 206 (100), 205 [M–H] ^+^ (21), 190 [M–OH] ^+^ (24) and 176 [M–2CH_3_] ^+^ (11); Analysis: calcd. %C, 64.08; %H, 4.83; %O, 31.88. Found: %C 64.12; %H, 4.81; %O 31.09.

### 3.7. 2,2-Dimethyl-2H-chromene-5,7,8(6H)-trione (**7**)

Compound 7 was obtained by the same procedure described for the synthesis of compound **4** as faint brown crystals (0.59 g, 40% yield) with mp 187–189 °C; IR (KBr) *ν*_max_ cm^−1^: 1,750, 1,720, 1,711, 1,581, 1,345, 1,099, 901, 869; ^1^H-NMR (CDCl_3_): δ 6.82 (Ar-1H, d, *J =* 9.2 Hz, H-4), 5.84 (Ar-1H, d, *J =* 8.2 Hz, H-3), 4.07 (Ar-1H, s, H-6), 1.44(6H, s, 2CH_3_); ^13^C-NMR (CDCl_3_): δ 176.5 (C-8), 196.2 (C-5,7), 141.2 (C-8a), 117.4 (C-4a), 126.5 (C-3), 115.5 (C-4), 45.8(C-6),86.7 (C-2), 29.5 (2CH_3_, C-9,10); EIMS (C_11_H_10_O_4_) (% int.) *m/z* 206 (100), 178 [M–CO] ^+^ (34) and 176 [M–2CH_3_] ^+^ (27); Analysis: calcd. %C, 64.06; %H, 4.88; %O, 31.04. Found: %C 63.92; %H, 5.81; %O 30.89.

### 3.8. 4,4,8,8-Tetramethyl-4,8-dihydro-2H-dipyrano-benzo-1,2-dione (**8**)

Compound **8** was obtained by the same procedures described for the synthesis of compounds **2** and **3**, respectively, by refluxing compound **7** (0.01 mol) with 3-chloro-3-methylbut-1-yne (0.01 mol), followed by ring closure to give the di pyrano derivative in dry xylene for 8 h, then the precipitate obtained was filtered off and crystallized from a mixture of cyclohexane and ethanol give compound **8** as faint brown crystals (0.85 g, 40% yields) with mp 292–294 °C; UV (MeOH) *λ*_max_: 462 (log ε 3.75), 302 (log ε 3.97), 271 (log ε 4.47), 261 (log ε 4.43); IR (KBr) *ν*_max_ cm^−1^: 1,789, 1,754, 1,598, 1,375, 1,049, 911, 859; ^1^H-NMR (CDCl_3_): δ 6.75 (d, *J =* 8.2, Ar-1H, H-10), 6.48 (d, *J =* 8.3 Hz, Ar-1H, H-6), 5.89 (d, *J =* 9.2 Hz, Ar-1H, H-9), 5.84 (Ar-1H, d, H-5), 1.34 (12H, s, 4CH_3_); ^13^C-NMR (CDCl_3_): δ 179.4 (C-1), 176.6 (C-2), 173.2 (C-6b), 131.3 (C-9), 130.1 (C-2a), 127.5 (C-6a), 125.5 (C-5), 97.1 (C-10a), 88.9 (C-8), 88.5 (C-4), 29.5 (2CH_3_, C-13,14), 28.7 (2 CH_3_; C-11,12); EIMS (C_16_H_16_O_4_) (% int.) *m/z* 272 (100), 257 [M–1CH_3_]^+^ (31), 244 [M–CO]^+^ (17) and 242 [M–2CH_3_]^+^ (14); Analysis: calcd. %C, 70.54; %H, 5.95; %O, 23.50. Found: %C 70.32; %H, 5.81; %O 23.39.

### 3.9. 6,6,10,10-Tetramethyl-6,10-dihydro-2H-dipyrano[2,3-f:2',3'-h]chromen-2-one (**10**)

Wittig reaction was carried out as described in the discussion [6] using 0.01 mol of compound **8**. 30% Aqueous NaOH solution (30 mL) was added to the Wittig reaction product and the mixture was boiled for 3 h, cooled, acidified with ice cold hydrochloric acid, and the reaction mixture refluxed for another 2 h. The precipitate obtained after cooling was filtered off, washed with water and crystallized from dil. ethanol to give compound **10** through the intermediate **9** as pale brown crystals (1.24 g, 50% yield) with mp 295–296 °C; UV (MeOH) *λ*_max_: 211 (log ε 4.27), 282 (log ε 3.75) and 232 (log ε 3.97); IR (KBr) *ν*_max_ cm^−1^: 1,720, 1,711, 1,591, 1,345, 1,089, 901, 869; ^1^H-NMR (CDCl_3_): δ 7.75 (Ar-1H, d, *J =* 8.95 Hz, H-4), 6.88 (Ar-1H, d, *J =* 8.9 Hz, H-12), 6.84 (Ar-1H, d, *J =* 9.2 Hz, H-8), 5.97 (Ar-1H, s, H-3,11), 1.84 (3H, s, H-13,14,15,16); ^13^C-NMR (CDCl_3_): 151.9 (C-12b), 149.8 (C-8b), 148.9 (C-4b), 139.5 (C-4), 128.5 (C-11), 128.2 (C-7,12), 116.4 (C-8), 113.6 (C-3), 108.2 (C-8a), 105.3 (C-4a), 102.5 (C-12a), 86.5 (C-6,10), 28.6 (4 CH_3_;C-13,14,15,16) as lit. [6]; EIMS (C_19_H_18_O_4_) (% int.) *m/z* 310. (100), 292 [M–CO] ^+^ (32), 285 [M–1CH_3_] ^+^ (21) and 250 [M–4CH_3_] ^+^ (7); Analysis: calcd. %C, 73.54; %H, 5.85; %O, 20.64. Found: %C 73.32; %H, 5.81; %O 20.29.

### 3.10. Biological Activity

#### 3.10.1. Experimental Animals

Young adult (30 ± 5 g) ICR mice (half male and half female) were provided by the Egyptian Holding Company for Biological Products and Vaccines, Cairo, Egypt. Animals were maintained under standard conditions of ventilation, temperature (25 ± 2 °C), humidity (60%–70%) and light/dark condition (12/12 h). The rats were housed in stainless steel cages and provided with free access to food and drinking water *ad libitum*. 

#### 3.10.2. Effect of Compound **10** on the Tumor of S180-Bearing Mice

The effect of compound **10** solution on tumor growth was estimated by evaluating body weight, tumor weight, and percentage of tumor inhibition. S180 tumor cell line was originally obtained from Cairo Institute of Oncology, Cairo, Egypt and maintained as the ascites form by serial passages intraperitoneal in ICR mice. For solid tumor development, S180 cell suspension (0.2 mL, 2 × 10^7^ cells/mL) was inoculated subcutaneously into right armpits of mice under sterile condition. The mice were divided into six random groups (10 in each): S180-bearing control, normal control, compound 10 (50, 100, 250 and 500 mg/kg body weight). Test doses were decided on the basis of findings from preliminary studies. Body weight of animals was recorded before the experiment. The doses administrated p.o. daily for 12 days. Normal control and S180-bearing control groups received the same volume of normal saline. On the 13th day, all animals were euthanized. Their body and tumor weights were obtained and documented [11].

#### 3.10.3. Assessment of Humoral Immune Function: Quantitative Hemolysis of Sheep Red Blood Cells (QHS) Assay

The mice were injected i.p. with 3:5 (v/v) sheep red blood cells (SRBC, 0.2 mL) prepared in normal saline on the 8th day of the experiment. QHS assay was performed in those animals following the immunization. Eyeballs were removed and single cell suspensions of 1 × 10^6^/mL were prepared in phosphate buffer solution (PBS). A total of 1.0 mL of 0.4% SRBC and 1.0 mL of 10% guinea pig serum were mixed with cell suspension and incubated for 1 h at 37 °C. After a 3 min centrifugation at 3,000 rpm, the absorbance of the supernatant was measured at 413 nm using a spectrophotometer [12].

#### 3.10.4. Assessment of Cellular Immune Function

For the assessment of cellular immune function, lymphocyte proliferation and Natural Killer (NK) cell cytotoxicity tests were performed. After the experiment was completed, their spleens were aseptically removed and filtered over a double layer of stainless-steel mesh to obtain single cell suspension. After these washes in Hanks’ balanced salt solution, the spleen cells were finally suspended in 10% FCS RPMI 1640 media supplemented with benzyl penicillin 100 U/mL, streptomycin 100 μg/mL. The cell number was adjusted to 3 × 10^6^ cells/mL of culture media for subsequent experiments [12].

##### 3.10.4.1. Measurement of Lymphocyte Proliferation

For the splenocyte proliferation assay, the spleen cell suspension was added to micro plate wells with 5 μg/mL of concanavalin A (Con A, from *Canavalia ensiformis* Type III, Sigma, Munich, Germany) and a polyclonal T cell mitogen. The micro plates were cultured at 37 °C for 72 h in the humidified 5% CO_2_ incubator. At 72 h, 1 μ*Ci*/well ^3^H-TdR (thymidine, [methl-^3^H]) was added to each well. The cells were harvested 16 h later and the radioactivity incorporated was counted using a liquid scintillation counter [12].

##### 3.10.4.2. Evaluation of NK Cell Cytotoxicity

The splenocyte prepared as described above were used as effector cells. YAC-1 cells, mice lymphoma sensitive to NK cells were used as target cells. Effectors and target cells resuspended in RPMI-1640 medium supplemented with 3% heat-inactivated fetal bovine serum were added to each well of a 96-well U-bottom micro culture plate in triplicate to obtain an effectors/target (E/T) ratio of 50:1, and incubated at 37 °C in the humidified 5% CO_2_ incubator for 8 h. After centrifugation, the culture supernatants were admixed with lactate dehydrogenase (LDH) solution (100 μL/well) and the amount of released LDH was determined. The OD value of each well was measured at 490 nm using a spectrophotometer. The percentage of cytotoxicity generated by NK cells was calculated according to the following formula:

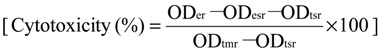
(1)
where OD_er_ (OD_experimental_ _release_) was the LDH release from co-cultures at an E/T ratio of 50:1; OD_esr_ (OD_effector_ _spontaneous_ _release_) and OD_tsr_ (OD_target_ _spontaneous_ _release_) were spontaneous LDH releases from effector and target cells incubated with medium alone, respectively; and OD_tmr_ (OD_target_ _maximum_ _release_) was the maximum release from target cells lysed with the lysis solution [13].

##### 3.10.4.3. Assessment of Nonspecific Immune Function: Phagocytic Activity of Macrophage

Phagocytic activity of macrophages was used to assess the nonspecific immune function. The mice were injected i.p. with 0.5 mL 5% cock red blood cells (CRBC) 10 h prior to the last dose. On the 13th day of the experiment macrophages were obtained from the peritoneal exudates harvested by peritoneal lavage using sterile cold Hanks’ solution. The number of CRBC ingested by macrophages was counted in an optical microscope [12].

### 3.11. Statistical Analysis

Data were expressed as the mean ± standard deviation (S.D.) in tables. Statistical analyses were carried out using the analysis of variance (ANOVA) and post-hoc tests for multiple comparisons. Differences were considered statistically significant at *p* < 0.05 [14].

## 4. Conclusions

In summary we have developed a new simple route approach to naturally dipetalolactone starting from resorcinol and substituted-1-butyne via Fremy’s salt as mediator oxidizing agent and examined its effect on *in vivo* Immunomodulatory activities in S180-bearing mice, where higher concentrations of compound **10** appeared to show more effective inhibition of tumor by increasing the antibodies secreted by spleen cells in mice. 
